# 39607 Mapping the Draining Lymph Nodes in Central Nervous System Malignancies

**DOI:** 10.1017/cts.2021.500

**Published:** 2021-03-30

**Authors:** Andrew T. Coxon, Barry A. Siegel, Tanner M. Johanns, Gavin P. Dunn

**Affiliations:** 1 Department of Neurological Surgery, Washington University School of Medicine; 2 Division of Nuclear Medicine, Mallinckrodt Institute of Radiology, Washington University School of Medicine; 3 Division of Medical Oncology, Washington University School of Medicine

## Abstract

ABSTRACT IMPACT: We seek to determine which lymph nodes drain the human brain. OBJECTIVES/GOALS: Lymphatic vessels train lymphatic fluid from the central nervous system (CNS), but the specific lymph nodes that these vessels drain to remains unknown in humans. We intend on using technetium tilmanocept (TcTM)to map the draining lymph nodes of the CNSin humans. METHODS/STUDY POPULATION: Patients having a tumor resected are eligible for the trial. All patients will have TcTM injected intracranially after tumor resection. Six patients will be enrolled in Cohort 1 to define the time course of drainage to the lymph nodes. Patients in Cohort 1 will be imaged with planar LS within 7 hours of injection and the following day. Either 12 or 24 patients will be enrolled into Cohort 2 to localize the draining lymph nodes with SPECT-CT. The optimal imaging timepoint from Cohort 1 will be used for Cohort 2. Patients in Cohort 2 will be stratified depending on if their tumor is in the frontal, parietal, occipital, or temporal lobe. RESULTS/ANTICIPATED RESULTS: We anticipate that we will detect TcTMin the deep cervical lymph nodes after injection into the brain. It is unclear exactly which lymph nodes the tracer will go to. We hypothesize that the results among patients will be similar, but interindividual variation is a possibility. Furthermore, patients with disease in different lobes of the brain may have different lymph drainage patterns. DISCUSSION/SIGNIFICANCE OF FINDINGS: We seek to answer a fundamental question of human anatomy: what lymph nodes drain the human brain? Additionally, knowing which nodes drain the human brain could shape future research of immunotherapy in patients with brain cancer or autoimmune disease such as multiple sclerosis.

